# Supplementary material for: Coated Hematite Nanoparticles Alleviate Iron Deficiency in Cucumber in Acidic Nutrient Solution and as Foliar Spray
Source: Plants (Basel). 2023 Aug 29;12(17):3104. doi: 10.3390/plants12173104 (PMC10490057; doi:10.3390/plants12173104)
Supplement: Supplementary file 1 [file plants-12-03104-s001.zip › plants-2571341-supplementary.pdf]

Coated hematite nanoparticles alleviate iron deficiency in cucumber in acidic nutrient solution and as foliar spray

Amarjeet Singh, Fruzsina Pankaczi, Deepali Rana, Zoltán May, Gyula Tolnai and Ferenc Fodor

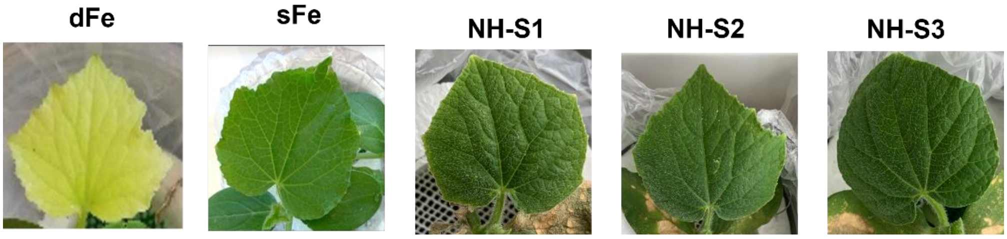

a)

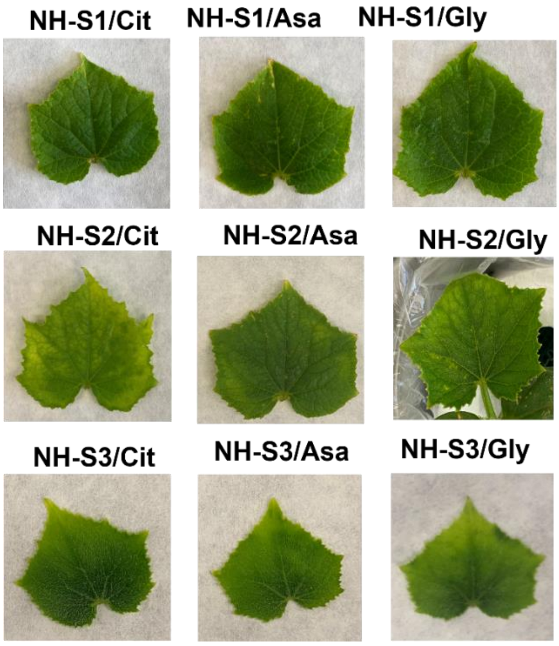

b)

Supplementary Figure 1. Pictures of representative 2nd leaf of plants harvested after 3 days **a)** showing control leaves and root supplied NHs and **b)** foliar treatment.
